# Expression of HSP70, IGF-1, OCT4, and AIF in Clear Cell Renal Cell Carcinoma

**DOI:** 10.3390/biomedicines14050974

**Published:** 2026-04-23

**Authors:** Matea Buljubašić Franić, Petar Todorović, Ivana Tica Sedlar, Natalija Filipović, Nela Kelam, Anita Racetin, Andrea Kopilaš, Ana Dunatov Huljev, Katarina Vukojević

**Affiliations:** 1 Department of Oncology, University Hospital Center Split, University of Split School of Medicine, 21000 Split, Croatia; mbuljubasic@kbsplit.hr (M.B.F.); itsedlar@kbsplit.hr (I.T.S.); 2 Department of Anatomy, Histology and Embryology, University of Split School of Medicine, 21000 Split, Croatia; petar.todorovic@mefst.hr (P.T.); natalija.filipovic@mefst.hr (N.F.); nela.kelam@mefst.hr (N.K.); anita.racetin@mefst.hr (A.R.); 3 Department of Family Medicine, Health Center Mostar, Bulevar Hrvatskih Branitelja b.b., 88000 Mostar, Bosnia and Herzegovina; andrea.kopilas@mef.sum.ba; 4 Department of Pathology, Forensic Medicine and Cytology, University Hospital Center Split, 21000 Split, Croatia; 5 Mediterranean Institute for Life Sciences, University of Split, 21000 Split, Croatia; 6 Center for Translational Research in Biomedicine, University of Split School of Medicine, 21000 Split, Croatia

**Keywords:** clear cell renal cell carcinoma, tumor grade, immunofluorescence, protein expression, HSP70, IGF-1, OCT4, apoptosis-inducing factor, tumor differentiation

## Abstract

**Background/Objectives**: Clear cell renal cell carcinoma is the most common subtype of kidney cancer and exhibits marked biological heterogeneity, even among tumors of the same histological grade. Although tumor grade remains a key prognostic parameter, the molecular alterations associated with tumor differentiation are not fully understood. This study aimed to evaluate grade-dependent tissue-level expression patterns of proteins involved in cellular stress response, growth regulation, stemness, and apoptosis in clear cell renal cell carcinoma. **Methods**: Protein expression of heat shock protein 70, insulin-like growth factor 1, octamer-binding transcription factor 4, and apoptosis-inducing factor were analyzed in human clear cell renal cell carcinoma samples and normal renal cortex. Low-grade and high-grade tumors were compared using immunofluorescence staining combined with semi-quantitative and quantitative image analysis. The proportion of positive signals and the number of positive cells were assessed across tissue compartments. In addition, publicly available transcriptomic data from The Cancer Genome Atlas kidney renal clear cell carcinoma cohort were analyzed to explore associations between gene expression levels and overall survival. **Results**: Distinct grade-dependent expression patterns were observed for all investigated proteins. Heat shock protein 70, insulin-like growth factor 1, and octamer-binding transcription factor 4 showed a higher expression in normal renal tissue with a progressive reduction across tumor grades. In contrast, apoptosis-inducing factor exhibited increased expression in tumor tissue, particularly in low-grade tumors, with a relative decrease in high-grade carcinomas. Stromal compartments of tumor tissue showed minimal or no expression for most markers. Transcriptomic survival analysis did not reveal significant differences in overall survival between high- and low-expression groups for any of the investigated genes. Grade-stratified transcriptomic analysis of the TCGA KIRC cohort revealed consistent patterns for HSP70 family members and OCT4, with progressive grade-dependent mRNA reduction toward higher grades, while IGF1 showed an inverse mRNA trend and AIFM1 showed a uniform reduction across all tumor grades without a clear inter-grade pattern. **Conclusions**: The findings demonstrate that stress response, growth-related, stemness-associated, and apoptotic proteins display distinct grade-dependent tissue-level expression patterns in clear cell renal cell carcinoma, with the expression profiles of high-grade tumors being of particular translational interest given the aggressive clinical behavior and therapeutic resistance characteristic of this disease stage. These alterations appear to reflect tumor differentiation and biological behavior rather than independent prognostic value, highlighting the complexity of molecular regulation in renal tumorigenesis.

## 1. Introduction

Renal cell carcinoma (RCC) represents the most common malignant tumor of the kidney in adults, accounting for approximately 85–90% of all primary renal neoplasms. Its incidence has steadily increased over recent decades, partly due to the widespread use of imaging techniques that enable the earlier detection of incidental renal masses [1]. Despite advances in diagnostic approaches and therapeutic strategies, RCC remains a clinically challenging disease characterized by pronounced biological heterogeneity, variable clinical behavior, and frequent resistance to therapy [2,3].

Clear cell renal cell carcinoma (ccRCC) is the most prevalent histological subtype, comprising approximately 70–75% of RCC cases [4]. It arises from epithelial cells of the proximal renal tubules and is characterized by distinct morphological and molecular features. The biological behavior of ccRCC is highly heterogeneous, ranging from indolent, low-grade tumors to aggressive high-grade carcinomas with invasive and metastatic potential [5]. Although histopathological parameters such as tumor grade and stage remain essential for clinical assessment, they do not fully capture the molecular complexity underlying ccRCC progression, highlighting the need for additional biological markers that may reflect tumor behavior more accurately [6]. A hallmark of ccRCC biology is dysregulation of cellular stress responses, apoptotic pathways, growth factor signaling, and mechanisms related to cellular plasticity and stemness. These processes allow tumor cells to adapt to hypoxic conditions, metabolic stress, and therapeutic pressure, thereby promoting survival and progression [7]. Consequently, proteins involved in these pathways have attracted increasing attention as potential markers of tumor differentiation, aggressiveness, and biological behavior [8].

Heat shock protein 70 (HSP70) is a molecular chaperone that plays a central role in maintaining protein homeostasis under physiological and stress conditions. In cancer, HSP70 has been implicated in cytoprotection, inhibition of apoptosis, and adaptation to hostile microenvironments [9,10]. In ccRCC, previous studies have reported altered HSP70 expression compared with normal renal tissue, although findings remain heterogeneous [11]. While some immunohistochemical studies describe reduced cytoplasmic HSP70 expression in higher-grade tumors, transcriptomic analyses suggest increased HSP70 gene expression associated with adverse clinicopathological features [11]. These discrepancies indicate that HSP70 regulation in ccRCC may be context-dependent and influenced by tumor grade, cellular localization, and methodological approach [12].

The insulin-like growth factor 1 (IGF-1) signaling axis represents another key pathway implicated in tumor growth and survival. IGF-1 mediates its effects through activation of the IGF-1 receptor and downstream signaling cascades such as PI3K/AKT and MAPK/ERK, which promote proliferation and inhibit apoptosis [13,14]. In RCC, components of the IGF system have been reported to be dysregulated, with evidence suggesting an association between IGF signaling and tumor progression [15]. However, the expression patterns of IGF-1 at the tissue level across different ccRCC grades remain insufficiently characterized, particularly in relation to a normal renal cortex [16].

Apoptosis-inducing factor (AIF) is a mitochondrial protein involved in caspase-independent programmed cell death. In addition to its role in apoptosis, AIF contributes to mitochondrial homeostasis and cellular redox balance [17]. Reduced AIF expression has been reported in RCC, and experimental studies have demonstrated that restoration of AIF expression in renal cancer cell lines induces apoptosis [18,19]. These findings suggest that downregulation of AIF may facilitate tumor cell survival by impairing intrinsic apoptotic mechanisms, potentially contributing to tumor progression and resistance to cell death in ccRCC [18,19].

Octamer-binding transcription factor 4 (OCT4) is a key regulator of pluripotency and self-renewal in embryonic stem cells and has been widely investigated as a marker of cancer stem cell-like phenotypes [20,21]. OCT4 exists as at least two functionally distinct isoforms: OCT4A, which localizes to the nucleus and regulates pluripotency-associated gene expression; and OCT4B, a cytoplasmic isoform implicated in cellular stress responses independently of stemness regulation [22]. In RCC, OCT4 expression has been detected in tumor tissues and linked to stemness-associated features, including enhanced proliferative capacity and resistance to stress [23,24]. Several studies have reported associations between OCT4 expression and unfavorable clinicopathological characteristics, suggesting that OCT4-positive tumor cell subpopulations may contribute to aggressive tumor behavior in ccRCC [25].

Despite growing interest in these molecules, comparative analyses of HSP70, IGF-1, OCT4 and AIF expression across different ccRCC grades, directly contrasted with normal renal tissue, remain limited. Furthermore, the relationship between tissue-level protein expression and transcriptomic survival data has not been comprehensively explored in a unified analytical framework.

Therefore, the present study aimed to evaluate the expression patterns of HSP70, IGF-1, OCT4, and AIF in clear cell renal cell carcinoma by comparing their spatial distribution, staining intensity, and proportion of positive cells in low-grade and high-grade ccRCC, as well as in normal renal cortex using immunofluorescence analysis. In parallel, transcriptomic data from the TCGA KIRC cohort were analyzed to assess potential associations between mRNA expression levels of the investigated markers and overall survival. By integrating tissue-based protein expression with large-scale transcriptomic analysis, this study seeks to characterize grade-dependent expression differences in these markers in an exploratory framework, with the ultimate aim of identifying molecular features of high-grade ccRCC that may inform future investigations into targeted therapeutic strategies.

## 2. Materials and Methods

### 2.1. Tissue Procurement and Processing

The present study was conducted as a retrospective analysis of archived formalin-fixed paraffin-embedded nephrectomy specimens. Ethics committee approval was obtained retrospectively for the immunofluorescence analysis of these archived tissue blocks in accordance with standard institutional practice for retrospective tissue-based research. The sample includes 17 patients with a pathological diagnosis of ccRCC (Table 1). Renal tissue paraffin blocks from nephrectomy were collected at the Department of Pathology, Forensic Medicine and Cytology at the University Hospital of Split from 2019 to 2020 and processed with the permission of the Ethical Committee of the University Hospital in Split (class: 500-03/23-01/04, class nr.:2181-147/01/06/LJ.Z.-23-02), following the Helsinki Declaration. Informed consent was obtained from all patients as part of the standard institutional procedure at the time of surgical intervention, covering the use of surgically resected tissue for research purposes. Clinical data from the time of the nephrectomy were extracted from the pathology report. Inclusion criteria required sufficient paraffin block material for immunohistochemistry (IHC) and complete clinical data. Exclusion criteria comprised incomplete laboratory results or insufficient material for IHC. Control tissue consisted of a normal renal cortex obtained from the non-tumoral part of the same nephrectomy specimen, distant from the tumor margin. Kidney tissue specimens were excised, individually fixed overnight in 4% paraformaldehyde (PFA), and dissolved in 0.1 M phosphate-buffered saline (PBS) in preparation for standard histological processing, including hematoxylin–eosin and immunofluorescence staining. After fixation, tissues were dehydrated through a series of ascending ethanol concentrations, embedded in paraffin, and sectioned serially at 4 µm thickness before being placed onto glass slides.

### 2.2. Immunofluorescence

Tissue sections were deparaffinized using xylol and rehydrated through a descending series of ethanol solutions. Heat-induced antigen retrieval was performed in 0.01 M citrate buffer (pH 6.0) at 95 °C for 30 min using a water steamer, after which sections were allowed to cool to room temperature. Following a wash step with 0.1 M PBS, nonspecific binding was blocked by applying a commercial protein-blocking solution (ab64226, Abcam, Cambridge, UK) for 20 min. Primary antibodies were then applied to the sections, which were incubated overnight in a humidified chamber. On the following day, slides were washed with PBS and subsequently incubated with the appropriate secondary antibodies (Table 1) for one hour. Nuclear counterstaining was performed using DAPI (4′,6-diamidino-2-phenylindole) after a final PBS rinse. Sections were then air-dried and coverslipped using Immuno-Mount (Thermo Shandon, Pittsburgh, PA, USA). Antibody specificity was verified through pre-adsorption controls, in which each primary antibody was diluted to its working concentration in blocking solution and pre-incubated with the corresponding peptide antigen before application to tissue sections; no immunoreactive signal was detected under these conditions. Additionally, omission of primary antibodies from the staining protocol produced no nonspecific secondary antibody binding or false-positive staining.

### 2.3. Data Acquisition and Statistical Analysis of the Area Percentage

Hematoxylin–eosin (H&E)-stained sections were imaged using a BX40 light microscope (Olympus, Tokyo, Japan). Immunofluorescence images of renal cortex tissue were acquired with a BX51 fluorescence microscope (Olympus, Tokyo, Japan) equipped with a Nikon DS-Ri2 digital camera (Nikon Corporation, Tokyo, Japan) and operated using NIS-Elements F software (version 3.0). For each marker, ten non-overlapping fields representative of the tissue were selected and photographed at ×40 magnification under standardized exposure settings. Positive immunoexpression of IGF-1 and AIF was identified as a green fluorescent signal, either diffuse or punctate, whereas HSP70 and OCT4 positivity was identified by a red fluorescent signal.

A semi-quantitative scoring system was applied to all four markers (HSP70, IGF-1, AIF, and OCT4). Staining intensity was classified as follows: (−) absent; (+) mild; (++) moderate; and (+++) strong. The distribution of positive signal was graded as: (n) negative, corresponding to fewer than 10% of epithelial or carcinoma cells stained; (f) focal, indicating positivity in 10–50% of neoplastic cells; and (d) diffuse, denoting staining in more than 50% of neoplastic cells.

Quantitative assessment of immunoreactivity was carried out using ImageJ software (version 1.51; NIH, Bethesda, MD, USA). The proportion of tissue area occupied by positive fluorescent signals was determined by applying median filter subtraction followed by color thresholding, in accordance with previously established protocols [26,27,28]. Background thresholds were independently established by three experienced histologists based on negative control images. All statistical analyses were conducted in GraphPad Prism 9.0.0 (GraphPad Software, San Diego, CA, USA), and statistical significance was defined as *p* < 0.05. Given the non-normal distribution of the data, results are presented as a median and interquartile range (IQR). All graphs were assembled using GraphPad Prism 9.0.0. Plates were created using Adobe Photoshop (Adobe, San Jose, CA, USA).

Three complementary quantitative parameters were assessed for each marker: the percentage of tissue area covered by positive fluorescent signal (area percentage), as determined by ImageJ-based thresholding analysis; the absolute number of immunopositive cells per visual field, as determined by manual cell counting; and the ratio of immunopositive cells to total cells per visual field, calculated by dividing the number of positive cells by the total number of DAPI-stained nuclei. These parameters reflect distinct aspects of protein expression, signal distribution across tissue area, absolute cellular positivity, and proportional cellular positivity. Using the Kolmogorov–Smirnov test, the normality of the variables HSP70, IGF-1, OCT4, and AIF was assessed. As none of the variables followed a normal distribution (all *p* < 0.001), the Kruskal–Wallis test was applied for further analyses. The study was designed a priori with three predefined group comparisons, control, low-grade (G1), and high-grade (G4) ccRCC, and subsequent descriptive statements regarding expression differences are qualitative in nature and reflect effect size rather than formal post hoc inference. Considering interobserver variability, three researchers independently examined the collected microphotographs. Interrater agreement was demonstrated by interclass correlation analysis, which provided a coefficient greater than 0.80, suggesting excellent agreement [29].

### 2.4. Transcriptomics

We exported the data for the RNA expression of the HSP70, IGF1, OCT4, and AIF genes from the UCSC Xena database (University of California Santa Cruz, http://xena.ucsc.edu/ (accessed on 12 December 2025)). Overall survival and expression of HSP70, IGF1, OCT4, and AIF (mRNA seq) data from the GDC TCGA Kidney Clear Cell Carcinoma (KIRC) study was downloaded and processed in Microsoft Excel (Microsoft Corp., Redmond, WA, USA). Overall, 533 patient samples were included in the survival analysis after data curation for double samples. Survival analysis based on expression quartiles, i.e., between the lowest and highest quartile for each gene, was conducted in Past 4.0 software [30]. For statistical analysis, the Kaplan–Meier method and log-rank test were used for survival length analysis.

In addition, grade-stratified mRNA expression analysis of HSPA1A, HSPA1B, HSPA1L, IGF1, POU5F1, and AIFM1 was performed using the UALCAN platform (ualcan.path.uab.edu, accessed on 30 March 2026), which enables interactive visualization of TCGA gene expression data stratified by tumor grade. Box plots representing transcript per million values across normal renal tissue and tumor grades G1 through G4 were generated.

## 3. Results

Clear cell renal carcinoma (ccRCC) originates from epithelial cells of proximal tubules. Different grades of ccRCC were studied; a total of 17 patients were included, comprising nine low-grade (G1) and eight high-grade (G4) cases, whose clinical and demographic characteristics are presented in Table 2.

**Figure 1:**
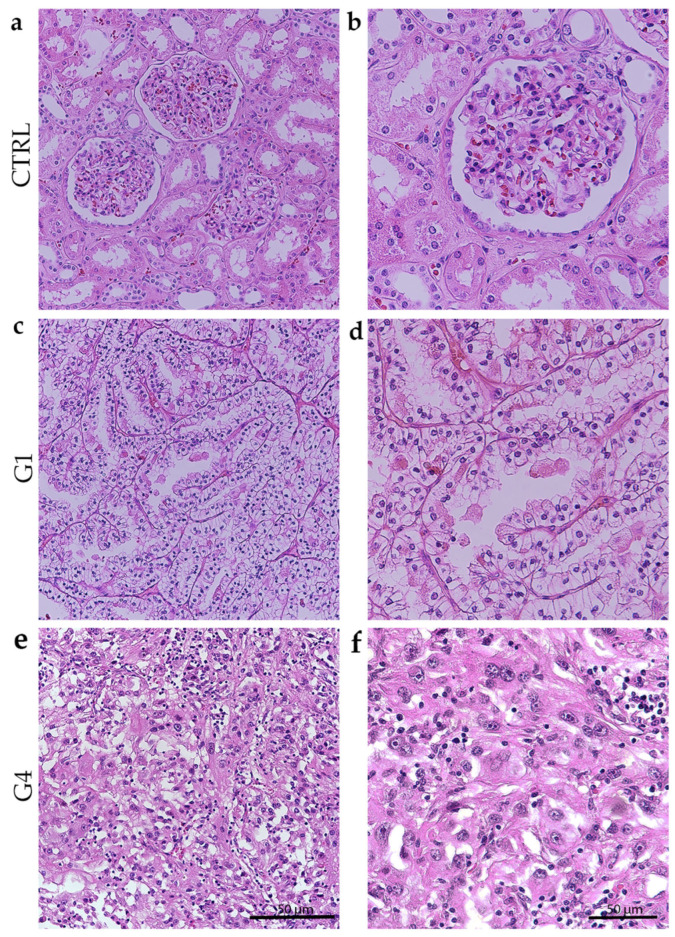
Hematoxylin and eosin staining of normal kidney cortex (CTRL) and different grades of renal clear cell carcinoma (ccRCC). Normal glomeruli and tubules (**a**); higher magnification of glomeruli and tubules in the renal cortex (**b**); clear cell carcinoma gradus 1-G1 low grade (**c**); higher magnification of ccRCC gradus 1 (**d**); ccRCC gradus 4-G4 (**e**); and higher magnification of ccRCC gradus 4 (**f**). Scale bar = 50 μm.

Low grade (grade 1) is characterized by small nuclei with inconspicuous nucleoli, while the highest grade (grade 4) is characterized by nuclear polymorphism and rhabdoid or sarcomatoid features (Figure 1).

**Figure 2:**
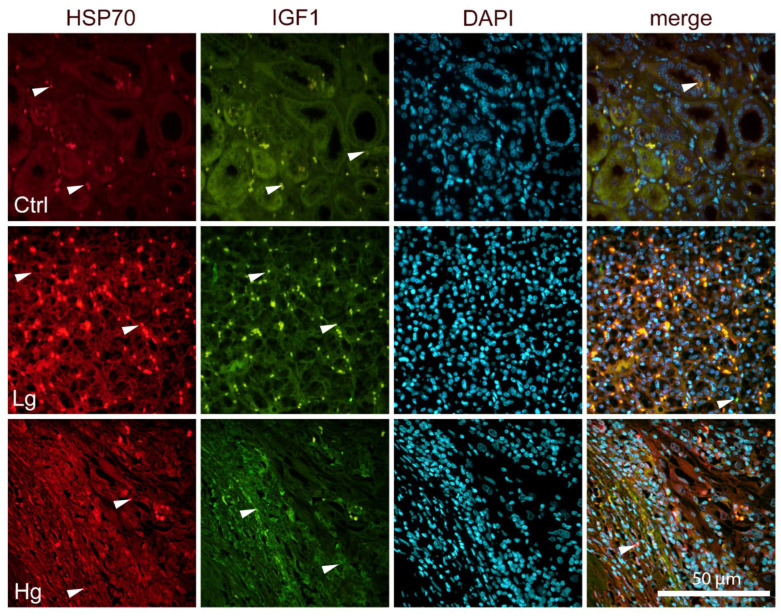
Immunofluorescence staining of HSP70 (red) and IGF-1 (green) in normal renal cortex (CTRL) and different grades of clear cell renal cell carcinoma (ccRCC). Normal glomeruli and tubules; low-grade ccRCC (G1); and high-grade ccRCC (G4). The first and second columns shows protein-specific fluorescent signal; the third column shows DAPI nuclear counterstaining (blue) enabling visualization of total cell number and tissue architecture; and the fourth column represents the merged overlay of all fluorescence channels allowing simultaneous visualization of protein expression and nuclear localization. Arrows indicate representative immunopositive cells regardless of staining intensity. Scale bar = 50 μm.

**Figure 3:**
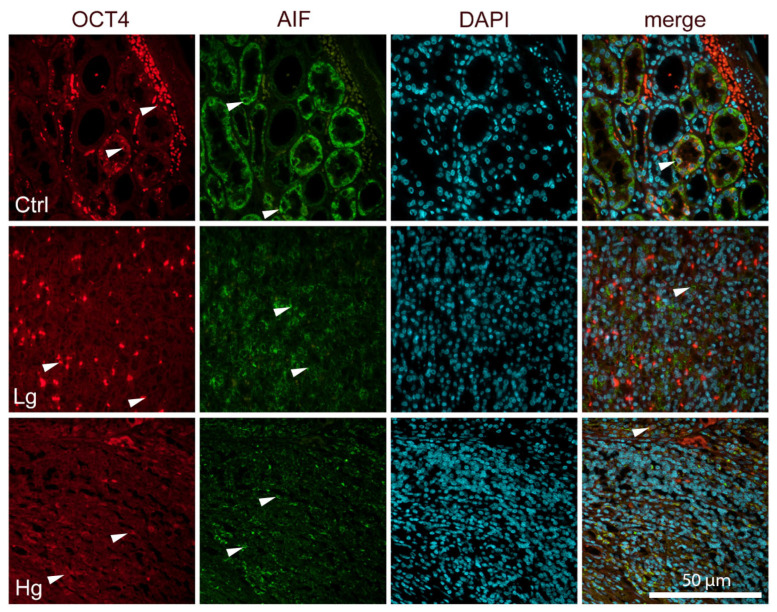
Immunofluorescence staining of OCT4 (red) and AIF (green) in normal renal cortex (CTRL) and different grades of clear cell renal cell carcinoma (ccRCC). Normal glomeruli and tubules; low-grade ccRCC (G1); and high-grade ccRCC (G4). The first and second column shows protein-specific fluorescent signal; the third column shows DAPI nuclear counterstaining (blue) enabling visualization of total cell number and tissue architecture; and the fourth column represents the merged overlay of all fluorescence channels allowing simultaneous visualization of protein expression and nuclear localization. Arrows indicate representative immunopositive cells regardless of staining intensity. A statistically significant difference in the expression parameters of HSP70, IGF-1, OCT4, and AIF was observed among the studied groups (controls, low-grade and high-grade ccRCC). For HSP70, the highest values were detected in the control group, followed by the low-grade group, with the lowest values observed in the high-grade group. A similar distribution pattern was observed for IGF-1 and OCT4, with the highest expression in controls, intermediate values in low-grade tumors and the lowest expression in high-grade tumors. In contrast, AIF expression was highest in the low-grade group, followed by the high-grade group, while the lowest values were observed in the control group. All differences were statistically significant (*p* < 0.001 for HSP70, IGF-1 and OCT4; *p* = 0.003 for AIF). Scale bar = 50 μm.

In the renal cortex of normal healthy kidneys, HSP70, IGF-1, OCT4, and AIF are ex-pressed occasionally in both glomeruli and tubules, as well as in high-grade and low-grade ccRCC (Figure 2 and Figure 3). HSP70 expression was highest in the control group, as were IGF-1 and OCT4, except for AIF, which was highest in the LG group of ccRCC (Table 3).

**Figure 4:**
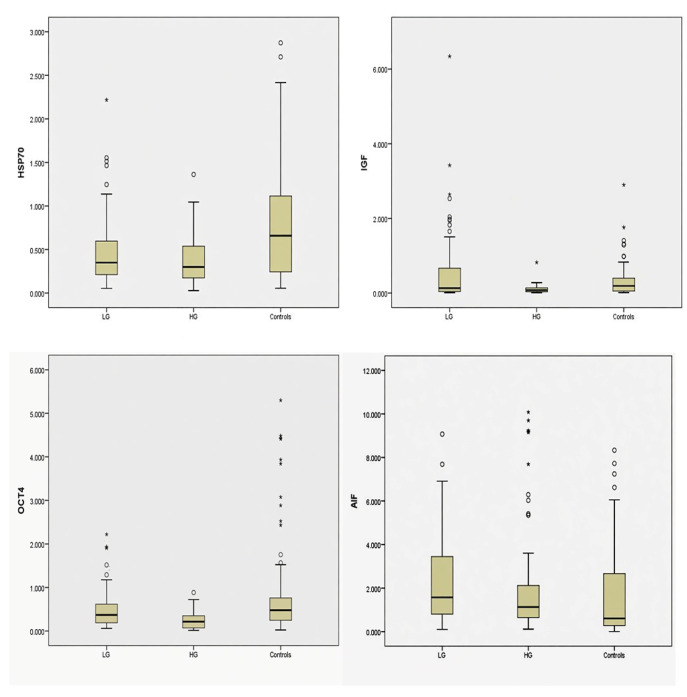
Box plots of parameter levels across groups. The percentage of signal expression is shown for low-grade (LG), high-grade (HG) tumors, and controls. The median is indicated by the central line, boxes represent the interquartile range (IQR), and whiskers denote the range. Circles indicate outliers. Asterisks (*) denote statistically significant differences between groups (* *p* < 0.05).

Figure 4 presents box plots illustrating the percentage of signal expression in control samples, low-grade tumors, and high-grade tumors. HSP70 showed the highest expression in the control group, with a median area percentage of 0.66 (IQR 0.88), whereas lower levels were observed in both low-grade (median 0.35, IQR 0.39) and high-grade tumors (median 0.30, IQR 0.37; Table 3). The expression of HSP70 in the control group was significantly higher than in both tumor groups (*p* < 0.001).

IGF-1 exhibited comparable median expression across all examined groups, with a median area percentage of 0.13 (IQR 0.63) in both the control group and low-grade tumors. High-grade tumors showed the smallest variability in IGF-1 expression, with a median of 0.08 and a markedly reduced IQR of 0.11 (Table 3), suggesting a more uniform reduction in signals in this group (*p* < 0.001). Similarly, OCT4 expression was highest in control samples (median area percentage 0.48, IQR 0.53), followed by low-grade tumors (median 0.37, IQR 0.43), and was lowest in high-grade tumors (median 0.21, IQR 0.28; Table 3). These differences were statistically significant (*p* < 0.001).

AIF showed relatively high expression across all examined groups, with the highest median area percentage observed in low-grade tumors (1.57, IQR 2.66), followed by high-grade tumors (1.13, IQR 1.48), and the lowest in controls (0.60, IQR 2.42; Table 3). The differences were statistically significant (*p* = 0.003).

The absolute number of immunopositive cells per visual field was analyzed in control samples, low-grade tumors, and high-grade tumors as a complementary parameter to the area percentage analysis presented above, revealing statistically significant differences among all groups (*p* < 0.001; Table 4). The median number of HSP70-positive cells was 93.5 in the control group, compared with 27.5 in high-grade tumors and 12 in low-grade tumors (*p* < 0.001).

A significantly higher number of IGF-1-positive cells was observed in the control group, with a median value of 156, compared with 23.5 in low-grade tumors and 43 in high-grade tumors (*p* < 0.001). For OCT4, the median absolute number of immunopositive cells per visual field was 55 in control samples and 41 in high-grade tumors, both of which were significantly higher than in low-grade tumors (17 positive cells; *p* < 0.001). These values represent the absolute cell counts per analyzed field and should be interpreted in conjunction with the area percentage data presented in Table 3, which confirmed the highest OCT4 signal in controls (median 0.48%), intermediate levels in low-grade tumors (median 0.37%), and the lowest expression in high-grade tumors (median 0.21%).

In contrast, AIF expression was lowest in the control group, with a median of 133.5 positive cells, whereas markedly higher numbers of AIF-positive cells were observed in both low-grade (median 372, IQR 286) and high-grade tumor groups (median 416, IQR 204; Table 4).

To further characterize the proportion of immunopositive cells relative to total cellularity, the ratio of positive to total cells was additionally analyzed across all groups (Table 5).

For HSP70, the median proportion of positive cells was highest in controls (0.40, IQR 0.18), markedly reduced in low-grade tumors (0.02, IQR 0.02) and in high-grade tumors (0.04, IQR 0.02; *p* < 0.001). For IGF-1, controls showed the highest proportion (0.57, IQR 0.31), with substantially lower values in low-grade (0.08, IQR 0.28) and high-grade tumors (0.07, IQR 0.06; *p* < 0.001). For OCT4, the proportion of positive cells was highest in controls (0.20, IQR 0.24), with lower values in low-grade (0.04, IQR 0.07) and high-grade tumors (0.06, IQR 0.04; *p* < 0.001). In contrast, AIF showed the highest proportion of positive cells in low-grade tumors (0.74, IQR 0.15), followed by high-grade tumors (0.71, IQR 0.13), while controls exhibited the lowest values (0.58, IQR 0.22; *p* < 0.001).

Differences in the intensity and distribution of immunofluorescent staining for HSP70, IGF-1, OCT4, and AIF were observed among control kidney tissue, low-grade ccRCC, and high-grade ccRCC (Table 5). Distinct expression patterns were noted between the examined groups. No staining or expression of HSP70, IGF-1, OCT4, or AIF was detected in the stromal component of either low-grade or high-grade tumors.

In low-grade ccRCC carcinoma cells, HSP70 and OCT4 showed no detectable expression, IGF-1 exhibited mild expression, while AIF demonstrated strong focal staining. In high-grade ccRCC carcinoma cells, AIF showed moderate expression with a diffuse staining pattern (>50% of neoplastic cells), whereas OCT4 displayed mild diffuse expression and IGF-1 showed mild focal expression.

In the control kidney, stromal and/or inflammatory cells exhibited absent to mild staining for HSP70 and IGF-1, mild focal staining for OCT4, and moderate diffuse staining for AIF. In contrast, epithelial cells of the control group demonstrated absent to mild OCT4 staining, moderate diffuse expression of HSP70 and AIF, and strong focal expression of IGF-1 (Table 6).

The association between survival and the expression levels of HSP70 (HSPA1A, HSPA1B, HSPA1L), IGF-1, AIF-1, and OCT4 (POU5F1) was evaluated in patients with clear cell renal cell carcinoma (ccRCC) by stratifying cases into high- and low-expression groups.

**Figure 5:**
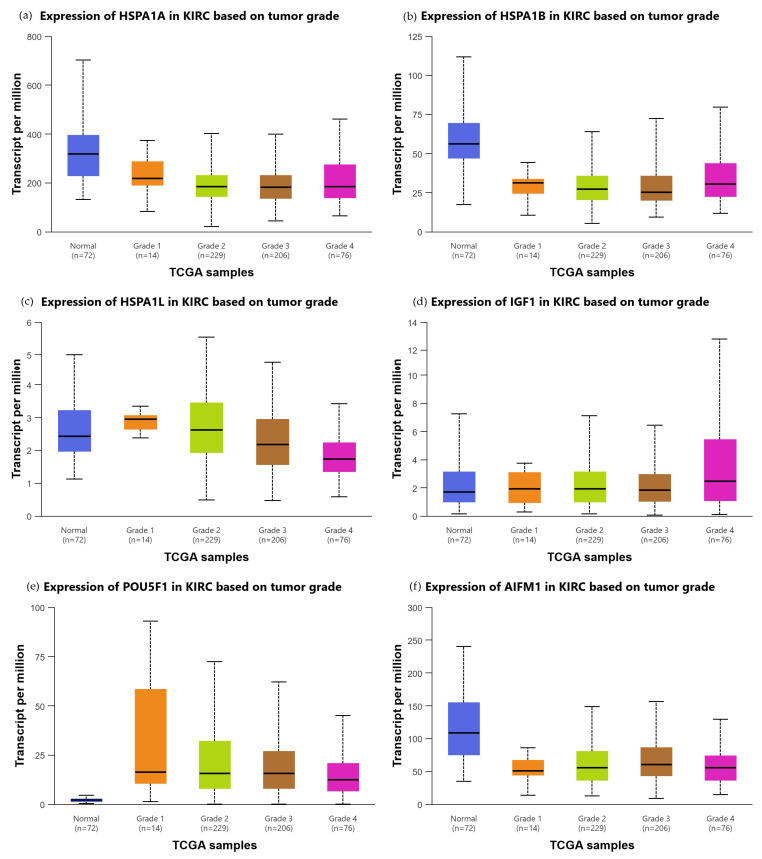
Grade-stratified mRNA expression of HSPA1A (**a**), HSPA1B (**b**), HSPA1L (**c**), IGF1 (**d**), POU5F1 (**e**), and AIFM1 (**f**) in TCGA Kidney Renal Clear Cell Carcinoma (KIRC) cohort. Box plots represent transcript per million (TPM) values across normal renal tissue (*n* = 72) and tumor grades G1 (*n* = 14), G2 (*n* = 229), G3 (*n* = 206), and G4 (*n* = 76). Statistical significance was assessed by unpaired two-sample *t*-test as implemented in the UALCAN platform. Data were retrieved and visualized using the UALCAN platform (ualcan.path.uab.edu).

Grade-stratified mRNA expression analysis of the investigated markers in the TCGA KIRC cohort is presented in Figure 5.

For HSPA1A, mRNA expression was significantly higher in normal renal tissue compared with grade 1 (*p* < 0.001), grade 2 (*p* < 0.001), and grade 4 (*p* < 0.001) tumors, with no significant differences between individual tumor grades. For HSPA1B, significant reductions were observed in all tumor grades compared with normal tissue (*p* < 0.001 for all), with additional significant inter-grade differences between grade 1 and grade 3 (*p* = 0.049) and grade 1 and grade 4 (*p* = 0.032). For HSPA1L, the most pronounced grade-dependent pattern was observed among HSP70 family members, with significant reductions in grade 3 (*p* = 0.010) and grade 4 (*p* < 0.001) compared with normal tissue, as well as multiple significant inter-grade differences including grade 1 vs. grade 3 (*p* = 0.0002), grade 1 vs. grade 4 (*p* < 0.001), grade 2 vs. grade 3 (*p* < 0.001), grade 2 vs. grade 4 (*p* < 0.001), and grade 3 vs. grade 4 (*p* = 0.018), collectively supporting a progressive grade-dependent reduction in HSPA1L expression. For IGF1, mRNA expression was significantly higher in grade 1 compared with normal tissue (*p* = 0.024), with a subsequent progressive increase toward higher grades, as evidenced by significant differences between grade 1 and grade 2 (*p* = 0.048), grade 1 and grade 3 (*p* = 0.029), grade 1 and grade 4 (*p* < 0.001), and grade 2 and grade 4 (*p* = 0.013), indicating an inverse grade-dependent mRNA trend compared with the protein-level reduction observed in the present immunofluorescence analysis. For POU5F1, mRNA expression was significantly higher in all tumor grades compared with normal tissue (*p* < 0.001 for all), consistent with the known induction of POU5F1 via a kidney-specific cryptic promoter in ccRCC, with significant inter-grade differences between grade 2 and grade 4 (*p* = 0.037) and grade 3 and grade 4 (*p* = 0.032), indicating a progressive decline at higher grades. For AIFM1, mRNA expression was significantly higher in normal renal tissue compared with all tumor grades (*p* < 0.001 for all), with no significant differences between individual tumor grades, indicating a uniform reduction in AIFM1 mRNA in tumor tissue without a clear grade-dependent inter-tumor pattern.

**Figure 6:**
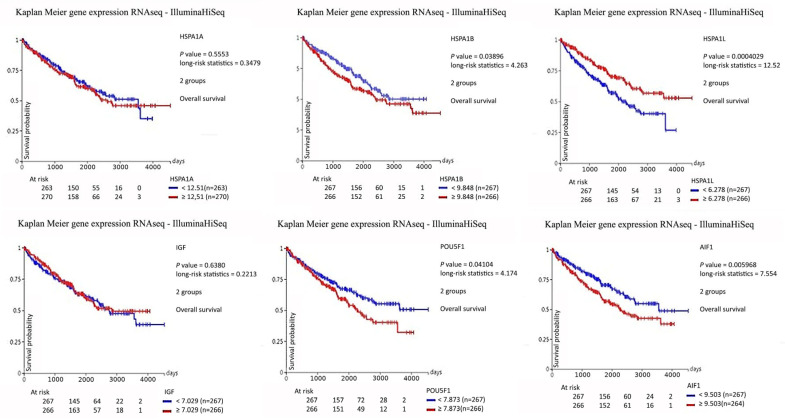
Graphic presentation of survival analysis (days) of HSP70 (HSPA1A, HSPA1B, HSPA1L), IGF, AIF 1, and OCT 4 (POU5F1) in high (blue line) and low (red line) mRNA expression in ccRCC is expressed as the average survival time in days. The KM method and log-rank test for survival length are used. Data are used from the GDC TCGA Kidney Clear Cell Carcinoma (KIRC) study.

Overall survival was assessed using Kaplan–Meier analysis, and the average survival time was calculated for each expression group (Figure 6).

Across all analyzed markers, no statistically significant differences in overall survival were observed between patients with high versus low expression levels, indicating that protein expression of HSP70, IGF-1, AIF-1, and OCT4 was not associated with survival outcomes in this cohort.

## 4. Discussion

Clear cell renal cell carcinoma (ccRCC) is a biologically heterogeneous malignancy characterized by complex alterations in cellular stress responses, apoptotic regulation, growth factor signaling and cellular plasticity [2,3]. Although the tumor grade remains one of the most important histopathological parameters for clinical stratification, the molecular mechanisms underlying grade-dependent differences in tumor behavior are incompletely understood [4,5]. Consequently, investigation of molecular pathways associated with tumor differentiation may provide additional insight into ccRCC biology beyond conventional histopathological assessment [8].

Proteins involved in stress response, growth factor signaling, stemness, and apoptosis have been increasingly implicated in renal carcinogenesis and tumor progression [7]. However, reported expression patterns of these molecules in ccRCC remain heterogeneous and, in some cases, contradictory, reflecting differences in study design, tumor grade distribution, analytical methods, and tissue compartment analysis [11,16,18]. Careful evaluation of protein expression at the tissue level therefore remains essential for improving understanding of ccRCC biology [6,8].

Among the investigated markers, the HSP70 immunofluorescence signal demonstrated a clear grade-dependent distribution, being highest in the control renal cortex, followed by low-grade ccRCC, with the lowest values observed in high-grade ccRCC. In addition, semi-quantitative compartment-based assessment indicated no detectable staining in the stromal/inflammatory compartment of either low- or high-grade tumors (Table 5).

This pattern is consistent with the findings of Ramp et al., who analyzed HSP70 protein expression in a large cohort of 145 clear cell RCCs and concluded that nuclear HSP70 immunoreactivity was present in all tumors, whereas cytoplasmic staining was detected in ~75%. Importantly, they reported significantly reduced cytoplasmic and combined nuclear/cytoplasmic HSP70 expression in ccRCC compared with non-neoplastic tubular epithelium, as well as a significant decrease in nuclear HSP70 expression from well- to poorly differentiated tumors, while no correlation with patient survival was observed [11]. The alignment between these results and our tissue-level observation of a higher HSP70 signal in control kidney and a lower expression in higher-grade ccRCC reinforces the notion that HSP70 protein undergoes grade-dependent downregulation at the tissue level.

A divergent picture emerges, however, at the transcriptomic level. Abd El-Fadeal et al. investigated HSP70 (HSPA4) mRNA expression and concluded that HSPA4 is upregulated in RCC and is positively associated with adverse clinicopathological features (including higher grade), with lower overall survival in high-expression groups [31]. This apparent discrepancy can be attributed to several factors. Most importantly, the two studies investigated distinct members of the HSP70 family, as while the present immunofluorescence analysis targeted the inducible HSP70 protein encoded by HSPA1A and HSPA1B, Abd El-Fadeal et al. focused on HSPA4, a structurally and functionally distinct family member subject to different transcriptional and post-translational regulatory mechanisms. Furthermore, post-transcriptional regulation, differential protein stability, and subcellular compartmentalization can produce substantial divergence between mRNA abundance and tissue-level protein content, particularly under the metabolic and hypoxic stress conditions characteristic of the ccRCC microenvironment [12]. Bulk transcriptomic analysis does not discriminate between cytoplasmic and nuclear protein pools, whereas compartment-resolved immunofluorescence captures these distinct localizations separately, which may further contribute to the observed differences.

The functional relevance of specific HSP70 family members is further illustrated by the work of Singh and Suri, who focused on HSP70-2 (HSPA2) in RCC cell lines and demonstrated that HSP70-2 knockdown reduces cell growth, colony formation, migration, and invasion, supporting a pro-tumorigenic role for specific HSP70 family members in vitro [32]. Taken together, these findings, integrated with the present results, suggest that HSP70-related pathways in RCC are family member- and context-dependent, and that protein localization and tumor compartment analysis may capture distinct biological aspects compared with transcriptomic signatures.

Regarding IGF-1, a distinct grade-dependent expression pattern was observed, with protein levels being highest in control renal tissue and low-grade ccRCC, whereas a marked reduction was detected in high-grade tumors, as demonstrated by both percentage of signal expression and the number of IGF-1-positive cells. In addition, semi-quantitative compartment analysis revealed no detectable IGF-1 staining in the stromal component of either low- or high-grade ccRCC, indicating that IGF-1 expression in our material was restricted to epithelial and carcinoma cells.

These findings need to be interpreted in the context of the broader IGF-1 literature, which has primarily characterized IGF-1 as a pro-tumorigenic factor in RCC. Cheung et al. concluded that IGF-1 acts as a potent mitogen in renal cell carcinoma, demonstrating that exogenous IGF-1 significantly stimulates DNA synthesis in primary RCC cultures and in the metastatic RCC cell line SN12K1, while neutralization of IGF-1 or its receptor suppresses tumor cell growth [33]. Importantly, their study showed that IGF-1 expression and functional relevance were most pronounced in the metastatic RCC model, which suggests that the mitogenic role of IGF-1 may be more relevant in the context of advanced and metastatic disease than in primary tumor differentiation. The reduction in local IGF-1 protein observed in high-grade tumors in the present study may therefore not reflect loss of IGF-1 signaling activity per se, but rather a shift from autocrine ligand production toward increased responsiveness to paracrine or circulating IGF-1 sources, a mechanism that would not be captured by tissue-level immunofluorescence.

At the clinical and genetic level, Cao et al. reported that a functional IGF1 3′UTR polymorphism is associated with RCC susceptibility and prognosis, with genotypes linked to lower IGF1 expression and improved survival, while RCC patients exhibited higher circulating IGF-1 levels than controls [34]. This is particularly relevant in light of our findings, as it demonstrates that systemic and genetic regulation of the IGF axis may drive tumor behavior independently of local tissue-level protein expression, further supporting the interpretation that reduced intratumoral IGF-1 protein in high-grade ccRCC does not necessarily equate to reduced IGF-1 pathway activity.

The importance of distinguishing between ligand and receptor expression within the IGF axis is further underscored by the work of Parker et al., who concluded that tumoral IGF-IR expression is associated with worse cancer-specific survival, reporting an approximately 70% increased risk of ccRCC death in IGF-IR-positive tumors compared with IGF-IR-negative tumors (HR 1.7), with even higher risk estimates in cases with >50% IGF-IR expression [35]. The fact that receptor upregulation is associated with poor prognosis, while local ligand levels decrease with tumor grade in our cohort, raises the possibility that high-grade ccRCC cells may become increasingly dependent on receptor-mediated signaling driven by circulating or stromal IGF-1 rather than locally produced ligand. This ligand–receptor uncoupling within the tumor microenvironment represents a biologically plausible mechanism reconciling the apparent discrepancy between our tissue-level findings and the established pro-tumorigenic role of the IGF axis in advanced RCC.

Taken together, the present results demonstrate that local IGF-1 protein expression is preserved in normal renal cortex and low-grade ccRCC but undergoes progressive reduction with tumor dedifferentiation. In contrast, previous in vitro and clinical studies emphasize the importance of IGF-1 signaling activity, receptor expression, and circulating IGF-1 levels, particularly in advanced or metastatic RCC. The integration of these perspectives suggests that the IGF axis in ccRCC is characterized by a spatial and functional dissociation between intratumoral ligand production and systemic or receptor-driven signaling activity, the full implications of which warrant further investigation. Turning to OCT4, a progressive grade-dependent reduction in protein expression was observed, with the highest levels detected in control renal tissue and a stepwise decrease from low-grade to high-grade ccRCC, as demonstrated by both the percentage of signal expression and the number of OCT4-positive cells. Semi-quantitative analysis further revealed absence of OCT4 staining in the stromal compartment of both low- and high-grade tumors, indicating that OCT4 expression in our material was restricted to epithelial and carcinoma cells.

At first glance, this finding appears to contradict the established association between OCT4 and aggressive tumor behavior. Khan et al. concluded that metastatic RCC cell lines exhibit increased expression of stemness-associated genes, including OCT4, and demonstrated that OCT4-high subpopulations display enhanced clonogenic capacity and stem cell-like properties in vitro [36]. However, it is important to emphasize that their study was based on cell line models and gene expression profiling, rather than tissue-level protein analysis, and therefore reflects functional stemness in advanced or metastatic settings rather than histological tumor differentiation. The biological context of established metastatic cell lines differs substantially from that of primary tumor tissue, and transcriptomic stemness signatures in such models may not directly translate to immunofluorescence-detectable protein levels in graded primary ccRCC specimens. At the tissue level, Rasti et al. investigated OCT4 expression in a large RCC cohort using tissue microarrays and reported that high nuclear OCT4 expression, particularly in combination with NANOG, is associated with aggressive tumor behavior and worse progression-free survival, including in the ccRCC subtype [25]. This discrepancy with our results can be reconciled by considering several important methodological and biological distinctions. First, Rasti et al. specifically identified nuclear OCT4 localization as the prognostically relevant feature, whereas the present immunofluorescence analysis assessed total cellular OCT4 signal without subcellular compartment stratification. It is noteworthy in this context that the OCT4 immunofluorescence signal observed in the present study appeared predominantly cytoplasmic or mixed cytoplasmic/nuclear in distribution, rather than exclusively nuclear. This pattern is consistent with predominant expression of the OCT4B isoform, which localizes to the cytoplasm and is associated with cellular stress responses rather than transcriptional regulation of pluripotency genes, in contrast to the nuclear OCT4A isoform [22]. This is particularly relevant given that nuclear translocation of OCT4 is required for its function as a transcriptional regulator of stemness-associated genes, and cytoplasmic retention may therefore represent a functionally inactive pool of the protein. Furthermore, human proximal tubule cells, the cell of origin of ccRCC, have been shown to have a particularly low barrier to OCT4-mediated reprogramming compared with other somatic cell types, suggesting a tissue-specific context for baseline OCT4B expression in the kidney that may account for the signal observed in normal renal cortex in the present study [22,37]. Consequently, the bulk tissue-level signal detected in our material may predominantly reflect this cytoplasmic, functionally inactive fraction, while the nuclear OCT4 subpopulation identified as prognostically relevant by Rasti et al. may be present in only a small subset of cells that does not substantially contribute to the overall immunofluorescence signal. It is therefore plausible that overall protein levels decrease during dedifferentiation while a numerically small but functionally active nuclear OCT4-positive subpopulation retains prognostic significance. Second, cancer stem cell-like subpopulations with high OCT4 expression may constitute only a minor cellular fraction within high-grade tumors, insufficient to substantially alter bulk tissue-level immunofluorescence signal, yet capable of disproportionately driving clonogenic capacity and metastatic potential. Third, the co-expression of OCT4 with NANOG, emphasized by Rasti et al. as the key prognostic combination, was not assessed in the present study, and it is possible that the functional relevance of OCT4 in ccRCC is highly context-dependent, requiring co-activation of other stemness regulators to exert its pro-tumorigenic effects. Taken together, the present data indicate that OCT4 protein expression undergoes progressive reduction at the bulk tissue level with increasing tumor grade in ccRCC, a pattern that likely reflects the loss of differentiation-associated OCT4 activity during dedifferentiation rather than an expansion of stem cell-like properties. In contrast, previous studies emphasizing poor prognosis associated with OCT4 expression primarily focus on nuclear localization, co-expression with other stemness markers, or functional stem-like subpopulations, often assessed in advanced disease or experimental models. These perspectives are not mutually exclusive, as a global reduction in tissue-level OCT4 may coexist with the emergence of a small but aggressive OCT4-positive stem cell-like compartment, the detection of which requires subcellular resolution and co-marker analysis beyond the scope of the present study. Among the investigated markers, AIF displayed the most striking discrepancy compared with the existing literature, exhibiting higher expression in tumor tissue than in control renal cortex, with the highest levels observed in low-grade ccRCC, followed by high-grade tumors, while control renal tissue exhibited the lowest AIF signal. Quantitative analysis demonstrated statistically significant differences in both the percentage of AIF-positive cells (*p* = 0.003) and the absolute number of positive cells (*p* < 0.001). Semi-quantitative assessment further revealed strong focal AIF staining in low-grade carcinoma cells and moderate diffuse staining in high-grade tumors, whereas stromal and inflammatory compartments remained negative across all tumor grades.

This pattern stands in apparent contrast to the findings of Xu et al., who concluded that AIF is markedly downregulated in the majority of renal cell carcinomas compared with adjacent non-neoplastic kidney tissue. In their immunohistochemical and molecular analysis of RCC specimens, AIF loss was observed in more than 80% of cases and was attributed to chromosomal deletion and promoter methylation [19]. Importantly, the authors demonstrated that forced AIF expression in RCC cell lines induced massive apoptosis, mediated through interaction with the pro-apoptotic kinase STK3 (MST2), supporting a tumor-suppressive role of AIF in renal carcinogenesis.

In line with this, Wang et al. reported that reduced AIF expression in RCC is associated with increasing tumor grade and poorer postoperative survival. Their study showed that AIF expression was highest in normal renal tubules, progressively decreased across tumor grades, and that patients with AIF-negative tumors had significantly worse overall survival [18]. The authors proposed AIF as a potential diagnostic and prognostic biomarker for RCC progression.

Experimental evidence for the anti-tumor role of AIF was further provided by Wang et al., who demonstrated that AIF suppresses cell proliferation, induces apoptosis, and inhibits invasion and metastasis in ccRCC models. Using in vitro and in vivo approaches, the authors showed that AIF activation led to reduced tumor growth and metastatic potential, supporting the concept that AIF-mediated pathways restrain ccRCC aggressiveness [38].

The discrepancy between these findings and the present results warrants detailed mechanistic consideration. From a technical standpoint, differences between immunohistochemistry and immunofluorescence, including antibody specificity, antigen retrieval protocols, and signal quantification approaches, may contribute to divergent findings across studies. From a biological standpoint, the key to reconciling these observations lies in the dual functionality of AIF. Under physiological conditions, AIF resides within the inner mitochondrial membrane as a flavoprotein oxidoreductase that contributes to the assembly and stability of the mitochondrial respiratory chain complex I, independently of its apoptotic function [17]. Only upon receipt of lethal stimuli does AIF undergo proteolytic cleavage, release from the mitochondrial membrane and nuclear translocation, where it mediates caspase-independent chromatin condensation and cell death. The elevated AIF immunofluorescence signal detected in tumor cells in the present study therefore most plausibly represents the mitochondrial, non-apoptotic pool of the protein, potentially reflecting the well-documented increase in mitochondrial mass and oxidative metabolic activity that characterizes early-stage ccRCC cells adapting to the tumor microenvironment. This interpretation reconciles increased total protein detection by immunofluorescence with the functional impairment of AIF-mediated apoptotic signaling described by Xu et al. and Wang et al., as chromosomal deletion and promoter methylation may selectively abrogate apoptotic AIF function without proportionally reducing total mitochondrial AIF protein content detectable by antibody-based methods [18,19]. Additionally, inter-cohort variability in the prevalence of AIF genomic alterations and differences in patient selection and tumor heterogeneity may further contribute to the observed discrepancy. Importantly, the relative decline of AIF expression in high-grade compared with low-grade tumors in the present cohort is directionally consistent with the grade-dependent reduction described in the literature, supporting the concept of progressive attenuation of AIF-associated pathways during tumor dedifferentiation.

Taken together, these findings highlight that AIF biology in ccRCC cannot be reduced to a simple gain or loss model. Rather, the present results suggest that AIF protein is dynamically regulated across tumor grades in a manner that reflects the interplay between its mitochondrial homeostatic role and its apoptotic function, with the balance shifting toward functional apoptotic impairment as tumors progress to higher grades. In contrast, previous studies describing AIF downregulation as a feature of RCC progression and poor prognosis have primarily assessed functional or genomic loss of AIF activity, which may not be fully captured by total protein immunofluorescence. These perspectives are complementary rather than contradictory and together emphasize the importance of distinguishing between total protein abundance and functional protein activity when interpreting tissue-level expression data in the context of ccRCC biology. The grade-stratified transcriptomic analysis of the TCGA KIRC cohort provides additional context for the tissue-level findings. For HSP70 family members, the progressive reduction in mRNA expression from normal tissue toward higher tumor grades, most pronounced for HSPA1L, is broadly consistent with the grade-dependent protein reduction observed in the present immunofluorescence analysis. For POU5F1, the elevated mRNA expression in tumor grades relative to normal tissue followed by a progressive decline toward grade 4 aligns with the grade-dependent protein reduction at the tissue level and is consistent with the known induction of POU5F1 via a kidney-specific cryptic promoter in early ccRCC. For IGF1, the inverse grade-dependent mRNA trend, with lowest expression in grade 1 and progressive increase toward grade 4, further supports the interpretation that IGF-1 protein reduction in high-grade tumors reflects post-transcriptional regulation or a shift toward receptor-driven signaling rather than transcriptional downregulation. For AIFM1, the uniform reduction in mRNA across all tumor grades without inter-grade differences complements rather than contradicts the elevated total AIF protein signal observed by immunofluorescence, which has been attributed to the non-apoptotic mitochondrial pool of the protein.

In summary, this study provides a comparative tissue-level analysis of HSP70, IGF-1, OCT4, and AIF expression in low-grade and high-grade clear cell renal cell carcinoma and normal renal cortex. The results demonstrate distinct, grade-dependent expression patterns across the investigated markers, highlighting the heterogeneous molecular landscape of ccRCC. While HSP70, IGF-1, and OCT4 showed higher expression in control tissue with progressive reduction in tumor grades, AIF exhibited an opposite pattern, with increased expression in tumor tissue, particularly in low-grade ccRCC, and a relative decline in high-grade tumors. Importantly, integration of tissue-based protein expression with transcriptomic survival analysis did not reveal significant associations between marker expression levels and overall survival, suggesting that the observed alterations primarily reflect tumor differentiation and biological behavior rather than independent prognostic value. Collectively, the observed discrepancies between tissue-level protein expression and previously published transcriptomic, functional and immunohistochemical data underscore the importance of methodological context, subcellular protein localization and the distinction between total protein abundance and functional activity when interpreting molecular marker studies in ccRCC. The present results may serve as a pilot framework for future larger-scale investigations encompassing all tumor grades, which would allow more comprehensive characterization of the continuous variation with these markers across the full grading spectrum of ccRCC.

The present study has several limitations. The cohort is relatively small (*n* = 17), and the pilot nature of the study limits generalizability. Only grade 1 and grade 4 tumors were included, restricting characterization of continuous expression changes across the full grading spectrum. Complete pathological staging data were unavailable for all cases, and future studies should incorporate full TNM staging information given that prognosis in ccRCC is jointly determined by grade and stage. Control tissue was derived from the non-tumoral portion of the same nephrectomy specimen, a standard but inherently limited approach, as peritumoral parenchyma may be subject to field effects. Although intratumoral grade heterogeneity is a well-documented feature of ccRCC, grade was assigned according to the highest-grade component per WHO/ISUP criteria; therefore, intratumoral heterogeneity in G4 tumors would tend to attenuate rather than amplify observed differences, supporting the robustness of the present findings. Finally, isoform-specific analysis of OCT4 was not performed; future studies employing isoform-specific antibodies or subcellular compartment-resolved quantification would allow more precise characterization of OCT4A and OCT4B contributions to the observed expression patterns.

## Figures and Tables

**Table 1:** Primary and secondary antibodies used in the study.

Antibodies	Name	Catalog Number	Host	Dilution	Source
Primary					
	Anti-HSP70 antibody	ab31010	Rabbit	1:500	Abcam (Cambridge, UK)
	Anti-IGF1 antibody	AF-291-NA	Goat	1:300	R&D Systems (Minneapolis, MN, USA)
	Anti-OCT4 antibody	963209	Rabbit	1:800	Millipore, Temicula, CA, USA (AB3209)
	Anti-AIF antibody	AF5824	Sheep	1:500	R&D Systems (Minneapolis, MN, USA)
Secondary					
	Anti-Goat IgG, Alexa Fluor^®^ 594	705-295-003	Donkey	1:400	Jackson Immuno Research Laboratories, Inc. (Baltimore, PA, USA)
	Anti-Rabbit IgG, Alexa Fluor^®^ 488	711-545-152	Donkey	1:400	Jackson Immuno Research Laboratories, Inc. (Baltimore, PA, USA)
	Anti-Sheep IgG, Alexa Fluor^®^ 488	715-545-150	Donkey	1:400	Jackson Immuno Research Laboratories, Inc. (Baltimore, PA, USA)

**Table 2:** Patients’ characteristics according to ccRCC grades. Data are presented as mean ± standard deviation (SD).

Histology and Grade	No. of Patients (*n*)	Age (Year)	Male/Female (*n*)
ccRCC Low grade (G1)	9	64 ± 11	7/2
ccRCC High grade (G4)	8	64 ± 8	7/1

**Table 3:** Using the Kolmogorov–Smirnov test, the normality of the variables HSP70, IGF-1, OCT4, and AIF was tested. The mentioned variables are not normally distributed (for all *p* < 0.001); therefore, the Kruskal–Wallis test was used in the analyses.

Variable	Controls	LG	HG	*p*-Value
HSP70, median (IQR)	0.66 (0.88)	0.35 (0.39)	0.3 (0.37)	<0.001
IGF, median (IQR)	0.13 (0.63)	0.13 (0.63)	0.08 (0.11)	<0.001
OCT4, median (IQR)	0.48 (0.53)	0.37 (0.43)	0.21 (0.28)	<0.001
AIF, median (IQR)	0.60 (2.42)	1.57 (2.66)	1.13 (1.48)	0.003

**Table 4:** Analysis of the number of positive cells in relation to the total number of cells in controls, high-grade tumors, and low-grade tumors.

Variable	Controls	LG	HG	*p*-Value
HSP70-positive, median (IQR)	93.5 (89)	12 (7)	27.5 (8)	<0.001
IGF-positive, median (IQR)	156 (152)	23.5 (116)	43 (29)	<0.001
OCT4-positive, median (IQR)	55 (45)	17 (13)	41 (20)	<0.001
AIF-positive, median (IQR)	133.5 (60)	372 (286)	416 (204)	<0.001

**Table 5:** Ratio of immunopositive cells to total cells in control kidney tissue (CTRL) and low-grade (LG) and high-grade (HG) ccRCC. Data are presented as median (IQR). Statistical analysis was performed using the Kruskal–Wallis test.

Variable	Controls	LG	HG	*p*-Value
HSP70-positive ratio, median (IQR)	0.40 (0.18)	0.02 (0.02)	0.04 (0.02)	<0.001
IGF-positive ratio, median (IQR)	0.57 (0.31)	0.08 (0.28)	0.07 (0.06)	<0.001
OCT4-positive ratio, median (IQR)	0.20 (0.24)	0.04 (0.07)	0.06 (0.04)	<0.001
AIF-positive ratio, median (IQR)	0.58 (0.22)	0.74 (0.15)	0.71 (0.13)	<0.001

**Table 6:** Intensity and extent of immunofluorescent staining for HSP70, IGF-1, OCT4, and AIF in control kidney tissue (CTRL) and in clear cell renal cell carcinoma (ccRCC) of different grades (low grade, G1; high grade, G4). Staining patterns are shown separately for epithelial/carcinoma cells and stromal/inflammatory infiltrate. Staining intensity was graded as: −, no expression; +, mild expression; ++, moderate expression; and +++, strong expression. The extent of staining was classified as: n, no staining (<10% of epithelial/carcinoma cells); f, focal staining (10–50% of neoplastic cells); and d, diffuse staining (>50% of neoplastic cells).

		HSP70	IGF-1	OCT4	AIF
CTRL	Epithelial cells	++/d	+++/f	+/n	++/d
	Stroma/inflammatory infiltrate	+/n	+/n	+/f	++/d
Low grade G1	Carcinoma cells	−/n	+/n	−/n	+++/f
	Stroma/inflammatory infiltrate	−/n	−/n	−/n	−/n
High grade G4	Carcinoma cells	−/n	+/f	+/d	++/d
	Stroma/inflammatory infiltrate	−/n	−/n	−/n	−/n

## Data Availability

All data and materials are available upon request.

## Abbreviations

The following abbreviations are used in this manuscript:

AIF	Apoptosis-Inducing Factor
ccRCC	Clear Cell Renal Cell Carcinoma
CTRL	Control
DAPI	4′,6-Diamidino-2-Phenylindole
GDC	Genomic Data Commons
H&E	Hematoxylin and Eosin
HR	Hazard Ratio
HSP70	Heat Shock Protein 70
HSPA1A/1B/1L	Heat Shock Protein Family A Members 1A, 1B, 1L
HSPA2	Heat Shock Protein Family A Member 2
HSPA4	Heat Shock Protein Family A Member 4
IGF-1	Insulin-Like Growth Factor 1
IGF-IR	Insulin-Like Growth Factor 1 Receptor
IHC	Immunohistochemistry
KIRC	Kidney Renal Clear Cell Carcinoma
KM	Kaplan–Meier
MAPK/ERK	Mitogen-Activated Protein Kinase/Extracellular Signal-Regulated Kinase
mRNA	Messenger Ribonucleic Acid
NIH	National Institutes of Health
OCT4	Octamer-Binding Transcription Factor 4
PBS	Phosphate-Buffered Saline
PFA	Paraformaldehyde
PI3K/AKT	Phosphoinositide 3-Kinase/Protein Kinase B
POU5F1	POU Class 5 Homeobox 1
RCC	Renal Cell Carcinoma
SD	Standard Deviation
STK3/MST2	Serine/Threonine Kinase 3/Mammalian Sterile 20-like Kinase 2
TCGA	The Cancer Genome Atlas
UCSC	University of California Santa Cruz
WHO	World Health Organization
3′UTR	3′ Untranslated Region
